# Access to eye health services among indigenous Australians: an area level analysis

**DOI:** 10.1186/1471-2415-12-51

**Published:** 2012-09-24

**Authors:** Margaret Kelaher, Angeline Ferdinand, Hugh Taylor

**Affiliations:** 1 Centre for Health Policy, Programs and Economics School of Population Health, Faculty of Medicine, Dentistry and Health Sciences University of Melbourne, 207 Bouverie st Parkville, Melbourne, 3010, Australia; 2 Indigenous Eye Health Unit School of Population Health, Faculty of Medicine, Dentistry and Health Sciences University of Melbourne, 207 Bouverie st Parkville, Melbourne, 3010, Australia

**Keywords:** Aboriginal and Torres Strait Islander, Indigenous, Eye, Cataract, Equity

## Abstract

**Background:**

This project is a community-level study of equity of access to eye health services for Indigenous Australians.

**Methods:**

The project used data on eye health services from multiple sources including Medicare Australia, inpatient and outpatient data and the National Indigenous Eye Health Survey.

The analysis focused on the extent to which access to eye health services varied at an area level according to the proportion of the population that was Indigenous (very low = 0-1.0%, low = 1.1-3.0%, low medium = 3.1-6.0%, high medium = 6.1-10.0%, high = 10.1-20.0%, very high = 20 + %). The analysis of health service utilisation also took into account age, remoteness and the Socioeconomic Indices for Areas (SEIFA).

**Results:**

The rate of eye exams provided in areas with very high Indigenous populations was two-thirds of the rate of eye exams for areas with very low indigenous populations. The cataract surgery rates in areas with high medium to very high Indigenous populations were less than half that reference areas. In over a third of communities with very high Indigenous populations the cataract surgery rate fell below the World Health Organization (WHO) guidelines compared to a cataract surgery rate of 3% in areas with very low Indigenous populations.

**Conclusions:**

There remain serious disparities in access to eye health service in areas with high Indigenous populations. Addressing disparities requires a co-ordinated approach to improving Indigenous people’s access to eye health services. More extensive take-up of existing Medicare provisions is an important step in this process. Along with improving access to health services, community education concerning the importance of eye health and the effectiveness of treatment might reduce reluctance to seek help.

## Background

Equitable and accessible health services form a key component of equitable health outcomes. Addressing inequities in health services is particularly important in eye health where the disadvantage of Indigenous Australians is unequivocal. The rate of low vision in Indigenous adults is 2.8 times rates in the general population and the rate of blindness 6.2 times higher than general population rates [1].

A core concept in equitable health care is distribution according to level of need for services, without regard to characteristics that do not inform these needs [2-4]. Inequity in access to ophthalmic services has been shown to affect populations disadvantaged by social class, age, geographic location and ethnic minority communities [5-7], and the link between eye disorders and social and economic disadvantage has also been strongly demonstrated in high- and low-income countries [6-9]. Within Australia, it has been demonstrated that rates of cataract surgery vary considerably, and that these variations were not necessarily consistent with differences in cataract prevalence [10-12]. In Indigenous adults, only 65% of those who experienced vision loss from cataract received surgery [1]. Cataract causes 37% of blindness in Indigenous Australians and blindness due to cataract occurs some 12 times more commonly than in mainstream populations. These patterns demonstrate a need to develop an understanding of how ophthalmic services in Australia as a whole are being accessed by Indigenous populations.

Our project examines equity of access to eye health services at a community level by examining the relationship between the percentage of Indigenous people living in an area, socioeconomic status and remoteness, access to ophthalmic and optometric services and the professionals that provide them.

## Methods

### Data

The data request was reviewed by Medicare Australia to ensure that the proposed use of the data was ethical and compliant with relevant legislation including the Information Privacy Principles under section 14 of Privacy Act 1988, Health Insurance Act 1973, National Health Act 1953, Health identifiers Act 2010 and Freedom of information Act. The provision of hospital data from State and Territories was similarly reviewed by the relevant data custodians including Corporate Data Management & Reporting; Information Management Section, Australian Capital Territory (request 2762); Health Demand and Performance Evaluation, New South Wales Health; Acute Care Information Unit, Northern Territory Department of Health and Families; Statistical Output, Health Statistics Centre, Queensland Health; Operations Division, South Australia Health; Health Information Provision, Victorian Department of Human Services (request 2601); Data Collection and Analysis - Statutory and Non-Admitted; Information Management and Reporting ( Request 3a_2009OP), West Australian Department of Health Service Review and Enhancement; Department of Health Services, Tasmania.

Relevant legislation for standards for information collection, storage, access, transmission, disclosure, use and disposal included the Commonwealth *Privacy Act 1988*, *Information Privacy Act 2000* and the *Health Records Act 2001* in Victoria, *Health Records and Information Privacy Act 2002 No 71* and *Privacy and Personal Information Protection Act 1998* in New South Wales, *Health Records (Privacy and Access) Act 1997* in Australian Capital Territory, the *Information Privacy Act 2009* in Queensland, *Personal Information Protection Act 1991* in Tasmania, *Information Act 2002* in Northern Territory, *Information Privacy Bill 2007* in Western Australia and *Information Privacy Principles 2009* in South Australia.

#### Eye health practitioners

Data on the geographic distribution of ophthalmology practices was obtained from the Royal College of Ophthalmologists (n = 1058) membership in 2008. Data on the geographic distribution of optometry practices was obtained from the 2008 electronic white pages (n = 6270). The number of practitioners is estimated to be equal to the number of offices. This will overestimate practitioners if offices are only visited and operated periodically.

#### Medicare Australia and outpatient data

Medicare data were obtained for services provided by optometrists and ophthalmologists (consultation codes: 10900, 10918, 10914, 10913, 10915, 10912 and 10916; eye exams: 104,105,106,107,108,109). Data covered the period from 2004/05-2007/08 (Medicare Australia, 2009). The utilisation data was broken down by age (0–4, 5–14, 15–24, 25–34, 35–44, 45–54, 55–64, 65–74, 75–84 and 85+ years) and statistical subdivision (SSD). The SSD is a general purpose geographical unit determined by the Australian Bureau of Statistics. In aggregate, SSDs cover Australia without gaps or overlaps. For the 2001 Census there were 207 SSDs defined throughout Australia. Medicare is available to people who reside in Australia, excluding Norfolk Island, if they hold Australian or New Zealand citizenship or have been issued with or applied for a permanent visa. Medicare provides access to free treatment as a public patient in a public hospital, free or subsidised treatment by practitioners and free treatment by providers who bulk bill.

Data on attendances at outpatient from eye clinics were also collected from each State and Territory by SSD. Data on paediatric attendances were not available from one region of Western Australia because of privacy concerns. Attendance at this clinic was estimated based on the ratio between adult and child hospital separations attendances in the rest of WA. The only complete year of data available for all states and territories was for 2007/08. Outpatient data do not include data on either the age or the Indigenous status of patients seen.

Each State and Territory was contacted to identify any other major programs that would not be captured using Medicare and hospitalisation data. While some small additional programs were identified in Queensland, there was no evidence of other major initiatives.

#### Inpatient data

Hospital inpatient data for cataract related Australian National Diagnosis Related Groups (Australian patient classification codes, or AN-DRGs) (Lens procedures, C16A; Lens procedures, same day C16B) for public and private hospitals were obtained from New South Wales, Victoria, Queensland, South Australia and Tasmania by SSD and Aboriginality.

The availability of inpatient data from Western Australia and the Northern Territory was limited because of concerns of privacy. Western Australian data was provided by procedure and region. Rates of use in each SSD were then estimated based on population size. Northern Territory data for eye procedures was provided at Territory level. The distribution of services across the Territory was then estimated using the overall distribution of hospital procedures.

Inpatient data does include an Indigenous identifier but this suffers from well-documented problems of under-enumeration [13]. The extent of this problem varies between states and territories. Recent Australian Institute of Health and Welfare (AIHW) reports suggest that inpatient identification of Indigenous status is around 89% [13]. However, the application of correction factors is not currently recommended. It should also be noted that identification of Indigenous status is considered unreliable for the Australian Capital Territory and Tasmania [13].

### Analysis

Population projections by age and part of the state for the whole population were obtained from SuperTABLE 4.3.1 Build 10 [14]. Population projections for the Indigenous population were obtained from Experimental Projections of Aboriginal and Torres Strait Islander Australians, Aboriginal and Torres Strait Islander Commission (ATSIC) Regions, 2001–2009 [15]. These were then mapped into the new Indigenous regions and then into SSDs using the CP2006SSD_IREG concordance file [16]. Projections were then converted into a multiplier to adjust 2006 Census counts for the Indigenous and non-Indigenous populations for the years before and after 2006.

Intercooled Stata v10 was used to conduct a panel poisson regression using SSDs as the unit of analysis. The dependent variables for the primary care analysis were eye exams provided by optometrists and ophthalmologists through Medicare and hospitals. The dependent variables in the hospital analysis were the supply of services for cataracts through private and public hospitals. The independent variables were year and the percentage of Indigenous people living in each area. The analyses were run adjusting for age, remoteness and area socioeconomic advantage, disadvantage and socioeconomic educational status. Remoteness was coded into categories (Major city, Inner and Outer Regional and Remote/VRemote). Socioeconomic data were based on the Socio-economic Indexes for Areas (SEIFA) [17]. The SEIFA is used to compare how disadvantaged an area is in relation to other areas in Australia in terms of people’s access to material and social resources and their ability to participate in society.

Rates of cataract surgery were also compared to the national average and World Health Organization (WHO) guidelines [18]. Most developed countries conduct around 4000 to 6000 cataract surgeries a year.(20) Preventable blindness associated with cataract is unusual at these levels of surgery [19]. WHO recommendations for cataract surgery rate in the developing world is 3000 per million.(19) We evaluate levels of cataract surgery both against what would be expected in Australia and what would be expected in the developing world in order to highlight disparities between Aboriginal and Torres Strait Islander and other Australians. The national average was calculated based on the following (Insertion of intra-ocular lens prosthesis (193), Intracapsular crystalline lens extraction (195), Extracapsular crystalline lens extraction by aspiration alone (196), Extracapsular crystalline lens extraction by phacoemulsification (197), Extracapsular crystalline lens extraction by mechanical phacofragmentation (198), Other extracapsular crystalline lens extraction (199), Other extraction of crystalline lens (200) and Other application, insertion or removal procedures on lens. Procedure rates and population data were obtained from the Australian Institute of Health and the Australian Bureau of statistics respectively [20,21]. The National average for cataract surgery rates were 8689 and 9072 per million in 2006/07 and 2007/08. The 2007/08 estimate is used in this report. National data, rather than data obtained from the states and territories, were used for the estimate of averages to enhance replicability. These data vary slightly because of differences in classification.

## Results

Table 1 shows that remoteness, age distribution and socioeconomic status varied with the percentage of Indigenous people living in each area. Areas with a high percentage of Indigenous people were more likely to be remote, had a higher proportion of young people and had lower socioeconomic status than communities with a low proportion of Indigenous people living in it.

**Table 1:** Area demographics by the percentage of Indigenous people living in the area

**Area Demographic characteristics**	**Percent Indigenous people living in the area**					
	**Very low (0-1%)**	**Low (1.1-3.0%)**	**Low medium (3.1-6.0% )**	**High medium (6.1-10.0%)**	**High (10.1-20.0%)**	**Very high (>20%)**
Remoteness						
Major city	45.30%	42.10%	11.90%	0.60%		
Inner regional	5.70%	45.40%	16.30%	18.40%	12.10%	2.10%
Outer regional	8.90%	29.20%	15.10%	17.20%	17.70%	12.00%
Remote			11.10%	22.20%	44.40%	22.20%
Very remote coastal				33.30%	33.30%	33.30%
Very remote inland					33.30%	66.70%
Age						
0-4	10.55%	10.64%	21.66%	18.55%	23.61%	15.60%
5-14	12.69%	11.08%	18.11%	16.51%	18.37%	16.52%
15-24	16.12%	11.20%	12.73%	13.09%	16.64%	12.18%
25-34	17.43%	17.31%	7.76%	11.13%	5.28%	11.93%
35-44	17.07%	17.21%	5.80%	7.79%	4.25%	10.47%
45-54	10.00%	13.87%	9.28%	3.45%	7.33%	8.77%
55-64	4.54%	8.14%	8.90%	9.03%	5.59%	7.69%
65-74	2.63%	5.18%	6.62%	9.85%	6.58%	7.78%
75-84	3.57%	0.17%	0.01%	0.12	0.02%	0.15%
85+	5.70%	45.40%	16.30%	18.40%	12.10%	2.10%
SEIFA scores						
Relative Advantage and Disadvantage	1030.14	996.20	970.64	941.54	947.00	963.09
Economic Resources	1024.97	1007.14	1003.91	975.65	978.03	981.94

### Eye health practitioners

**Figure 1:**
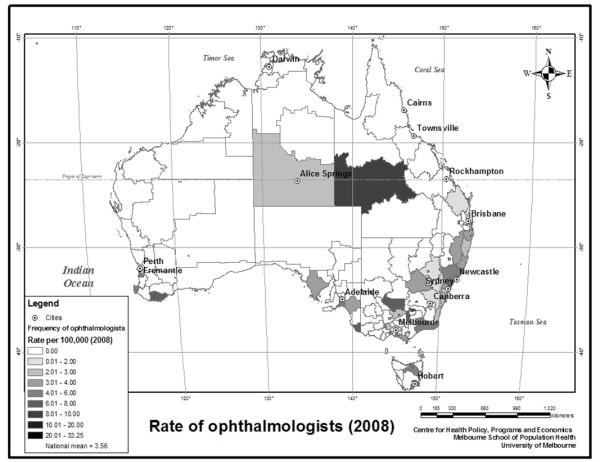
The frequency of ophthalmologist offices by SLA per 100,000 residents in 2008.

**Figure 2:**
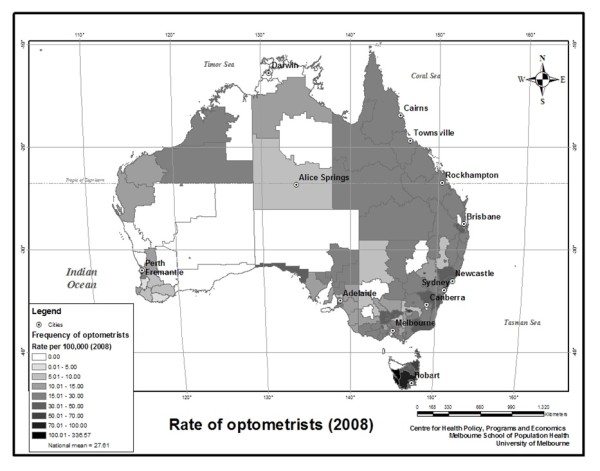
The frequency of optometrist offices by SLA per 100,000 residents in 2008.

Figures 1 and 2 show the distribution of ophthalmologists and optometrists throughout Australia by Statistical Local Area (SLA). Figure 1 shows that ophthalmologists are concentrated on the Eastern seaboard and metropolitan areas of Western Australia. However, there is also a high concentration of ophthalmologist offices in central Australia relative to the population. Optometrists tend to be far more numerous and evenly distributed than ophthalmologists.

### Eye exams

Table 2 shows that when all eye exams by optometrists and ophthalmologists are considered together there was still a significant disparity in the services provided in areas with low to high Indigenous populations compared to areas with very low Indigenous populations. The difference between areas was substantially reduced when socioeconomic status was added into the models. Differences in thelevel of eye exams became non-significant for areas with low to low medium Indigenous populations once remoteness and the socioeconomic position of the area were taken into account. Areas with high medium Indigenous populations had slightly higher levels of eye exams than areas with low indigenous population when all potential confounders were taken into account. Nonetheless the rate of eye exams was substantially lower in areas with very high Indigenous populations compared to areas with very low Indigenous populations.

**Table 2:** Poisson regression for eye exams in all sectors by optometrists and ophthalmologists in 2007/08

**Percent Indigenous**	**Incidence Rate Ratio (IRR) (95%CI)**	**IRR (95%CI) controlling for remoteness**	**IRR (95%CI) controlling for remoteness and SEIFA**	**IRR (95%CI) controlling for remoteness , SEIFA and age**
Very low (0-1%)	1.00	1.00	1.00	1.00
Low (1.1-3.0%)	0.97 (0.96-0.97)*	0.98 (0.97-0.98)*	0.99 (0.99-0.99)*	0.99 (0.99-0.99)*
Low medium (3.1-6.0% )	0.88 (0.88-0.88)*	0.91 (0.91-0.91)*	0.97 (0.97-0.97)*	0.98 (0.98-0.98)*
High medium (6.1-10.0%)	0.91 (0.9-0.91)*	0.99 (0.98-0.99)*	1.00 (1–1)*	1.03 (1.03-1.04)*
High (10.1-20.0%)	0.84 (0.83-0.84)*	0.92 (0.92-0.93)*	0.9 (0.89-0.9)*	1.0 (1.0-1.0)
Very high (>20%)	0.66 (0.65-0.66)*	0.79 (0.79-0.8)*	0.66 (0.65-0. 67)*	0.85 (0.83-0.85)*

*p < 0.05.

### Eye procedures

Table 2 shows that rates of cataract surgery in areas with high medium to very high Indigenous populations were less than half of those for reference areas. There was a trend for rates of cataract surgery to increase over time. However, this interacted with the Indigenous composition of the population such that rates of surgery were decreasing in areas with low and high to very high Indigenous populations compared to the reference area. Conversely, rates were increasing in areas where the Indigenous population was high medium compared to the reference areas.

A number of areas had cataract surgery below the levels generally recommended by the WHO to reduce cataract blindness in Africa. Around 40 percent of areas with very high Indigenous populations had cataract surgery rates below WHO recommended guidelines. This compares to around 6 percent in reference areas (Table 3). Around half of communities with very high and high medium Indigenous populations and over three quarters of communities with high Indigenous populations had rates of cataract surgery below the national average (see Table 4). The percentage of communities below the national average was 52 per cent in areas with very low Indigenous populations. In the remaining areas it was between 25 and 35 percent (see Table 4).

**Table 3:** Panel poisson regression for cataract surgery in all sectors by percent Indigenous population 2005/06-2007/08

	**IRR 95% CI#**
**Year**	
Linear trend	1.03 (1.02-1.04)*
**Percent Indigenous**	
Very low (0-1%)	1.00
Low (1.1-3.0%)	1.06 (1.01-1.11)*
Low medium (3.1-6.0% )	1.04 (0.97-1.11)
High medium (6.1-10.0%)	0.49 (0.41-0.58)*
High (10.1-20.0%)	0.34 (0.27-0.41)*
Very high (>20%)	0.42 (0.24-0.73)*
**Interactions**	
Year* Low (1.1-3.0%)	0.97 (0.96-0.98)*
Year* Low medium (3.1-6.0%)	1 (0.98-1.02)
Year* High medium (6.1-10.0%)	1.12 (1.09-1.15)*
Year* High (10.1-20.0%)	0.95 (0.91-0.98)*
Year* Very high (>20%)	0.95 (0.91-0.99)*

**p < 0.05, #Adjusted for age, remoteness and socioeconomic status.

**Table 4:** Areas where the 2007/08 cataract surgery rate is below WHO (3000 per million) and National Average (9072 per million)

**Percent Indigenous**	**Frequency 2005/06-2007/08**				
	**Below WHO guidelines**	**Below National Average**	**Total**	**Percent below WHO guidelines**	**Percent below National Average**
Very low (0-1%)	2	16	31	6.45	51.61
Low (1.1-3.0%)	0	22	62	0.00	35.48
Low medium (3.1-6.0% )	2	13	51	3.92	25.49
High medium (6.1-10.0%)	2	13	25	8.00	52.00
High (10.1-20.0%)	3	13	17	17.65	76.47
Very high (>20%)	8	11	22	36.36	50.00

## Discussion

The fragmented funding and service provision in the Australian health system creates a major challenge for health services research and the assessment of equity at a system level. We have drawn together information across sectors to provide a comprehensive assessment of eye health services for Indigenous Australians. The study supports the notion that socioeconomic status, ethnicity and geography can all contribute to inequalities in eye health [22-25]. It highlights the role of differential access health services in disparities in eye health between Indigenous and other Australians. The study also highlights the need to keep addressing disparities in eye health on the policy agenda [26].

As an ecological study, this research represents an indirect examination of Indigenous eye care access and utilisation. While there are other factors that affect this distribution, including measures of remoteness and socioeconomic disadvantage, there is little doubt that areas with a larger proportion of Indigenous Australians are the most disadvantaged in terms of access to eye health services at a primary care level. The rate of total eye exams provided in areas where the Indigenous population was very high was two-thirds of the rate of eye exams for areas where the Indigenous population was very low. Areas with very high Indigenous populations constituted about two-thirds of areas where the provision of eye health services was significantly below the national average.

Broadening the range of health professionals able to obtain reimbursement through Medicare is a key strategy of the reform of the Australian health system [27]. Optometrists were one of the first groups of health professionals other than doctors to be able to access Medicare. These data suggest that this strategy has benefits in terms of providing access to broader and more evenly distributed group of health professionals and may have reduced inequities in service utilisation. This is particularly true when age differences between areas are taken into account. There was also some evidence that eye problems may have to be severe before help is sought. This is reflected in many of the reasons provided in the National Indigenous Eye Health Survey for not having eye problems attended to [1]. As an indication, the most frequent reason provided is that the cataract is not severe enough to need surgery and the third most frequent reason in that the cataract is not bothersome [1]. This suggests that improving literacy around eye health may also be an important issue in improving the uptake of services.

It is concerning that many areas had rates of cataract surgery that fell below the WHO guidelines [28]. It is even more problematic that Indigenous communities were so strongly over-represented in this category. Over a third of areas with very high Indigenous populations and 17 percent of areas with high Indigenous populations had cataract rates below the minimum levels recommended by WHO. Around half of communities with very high and high medium Indigenous populations and over three quarters of communities with high Indigenous populations had rates of cataract surgery below the national average. However, around half of communities with very low Indigenous populations were also below the national average. The level of disadvantage experienced by Indigenous populations is more severe than for other Australians, although there was evidence that a high proportion of Australians are disadvantaged in relation to cataract surgery. It should be noted that the WHO standard is not age adjusted and accordingly neither are the comparator figures reported here. The WHO figures are based on African populations. Australia has a higher proportion of older people than most African countries. Accordingly it would be expected that age adjustment would reduce the performance of Australia relative to the standard. It may also decrease difference between Australian communities.

There is currently heated debate around reduction in the Medicare reimbursement rates for cataract and its implications for service provision [29]. Increases in the out-of-pocket costs for cataract surgery may contribute to increasing disparities among Indigenous and other Australians.

## Conclusions

The results suggest that despite a number of government initiatives to improve Indigenous people’s utilisation of eye health services there remain significant inequities in access. Even though Australia is a developed country, there was evidence that treatment for cataract in some areas with large Indigenous populations fell below WHO guidelines developed for Africa. Developing a targeted co-ordinated approach to address these issues is a challenge in an environment of complex service provision. More extensive take-up of existing Medicare provisions would be an important step in this process. The National Indigenous Eye Health Survey data suggest that along with improving access to health services, community education around the importance of eye health and the effectiveness of treatment might reduce reluctance to seek help.

## Abbreviations

ATSIC: Aboriginal and Torres Strait Islander Commission; AIHW: Australian Institute of Health and Welfare; SEIFA: Socioeconomic Indices for Areas; SLA: Statistical Local Area; SSD: Statistical subdivision; WHO: World Health Organization.

## Competing interests

The authors declare that they have no competing interests.

## Authors’ contribution

MK contributed to the design of the project, drafted the paper and conducted the final analysis. AF contributed to the design of the project, collected the data, conducted preliminary analysis and commented on drafts. HT contributed to the design of the project and commented on drafts. All authors read and approved the final manuscript.

## Pre-publication history

The pre-publication history for this paper can be accessed here:

http://www.biomedcentral.com/1471-2415/12/51/prepub
